# Controversies in the Management of Endometrial Carcinoma: An Update

**DOI:** 10.1155/2012/676032

**Published:** 2012-02-16

**Authors:** Mohamed K. Mehasseb, John A. Latimer

**Affiliations:** Department of Gynaecological Oncology, Addenbrooke's Hospital, Box 242, Hills Road, Cambridge, CB2 0QQ, UK

## Abstract

Endometrial carcinoma is the commonest type of female genital tract malignancy in the developed countries. Endometrial carcinoma is usually confined to the uterus at the time of diagnosis and as such usually carries an excellent prognosis with high curability. Our understanding and management of endometrial cancer have continuously developed. Current controversies focus on screening and early detection, the extent of nodal surgery, and the changing roles of radiation therapy and chemotherapy and will be discussed in this paper.

## 1. Introduction

 Endometrial carcinoma is the commonest type of female genital tract malignancy in the developed countries, accounting for nearly 50% of all new gynecologic cancers diagnosed in the Western world. Worldwide, endometrial cancer is only second to cervical cancer in frequency [1]. Most cases (75–85%) of endometrial carcinoma occur in the sixth and seventh decades of life, and 95% occur in patients over 40 years of age [2, 3]. 

Endometrial carcinoma is usually confined to the uterus at the time of diagnosis and as such usually carries an excellent prognosis with high curability [4, 5]. However, patients with high-risk factors including increased age, higher tumor grade, aggressive histology, and advanced stage represent real challenges.

Our understanding and management of endometrial cancer have continuously developed. The surgical approach has expanded to include pelvic and para-aortic lymphadenectomy, although debatable, and acceptance of laparoscopic management. There have been important developments in chemotherapy in endometrial cancer, which may be promising in an adjuvant setting [6]. Hormonal therapy remains an important option and our understanding of the biology of the disease may help determine which patients may benefit most [7]. Current controversies focus on screening and early detection, the extent of nodal surgery, and the changing roles of radiation therapy and chemotherapy and will be discussed in this paper.

## 2. Screening

Many endometrial cancers develop by way of a precursor lesion. Estrogen-related cancers frequently develop secondary to atypical endometrial hyperplasia (AEH) or demonstrate AEH in the uterus at the time of hysterectomy. Serous tumors also may develop through a precursor lesion, endometrial intraepithelial carcinoma (EIC) [8, 9]. Prompt identification of precursor lesions might potentially provide an opportunity to prevent cancers. However, mass screening of the population for endometrial cancer is not practical due to the low prevalence of the disease and as the ideal method for endometrial surveillance is yet to be devised [10, 11]. There is no blood test of sufficient sensitivity and specificity, and routine mass screening with pelvic ultrasound scans or endometrial biopsies is not practical. 

Malignant endometrial cells appear on Papanicolaou (Pap) smear in 25–50% of women with endometrial cancer [12–14]. However, this group is more likely to have deeper myometrial invasion, higher tumor grade, positive peritoneal cytologic findings, and a more advanced stage of disease at presentation [15]. The significance of normal endometrial cells in cervical smears in postmenopausal women is less clear. In asymptomatic postmenopausal women, the prevalence rate of (pre-)malignant uterine disease was significantly higher (6.5%) as compared to smears without these normal endometrial cells (0.2%) [16]. 

As there is no effective screening, management requires the prompt assessment of symptomatic patients, especially those at high risk. It is appropriate to evaluate individuals past their fourth decade of life if there is abnormal bleeding (i.e., intermenstrual bleeding, persistent blood stained discharge, postcoital bleeding). Similarly, a higher degree of suspicion should be held for younger patients with high-risk characteristics including significant obesity, polycystic ovarian syndrome/chronic anovulation, or tamoxifen exposure.

A meta-analysis reported that the Pipelle was the best endometrial sampling device, with detection rates for endometrial cancer in postmenopausal and premenopausal women of 99.6% and 91%, respectively. The sensitivity for the detection of endometrial hyperplasia was 81%. The specificity for all devices was 98% [17].

Screening and prevention strategies for women on tamoxifen are more challenging. Women with intact uteri who take tamoxifen for either treatment or prevention of breast cancer are at an increased risk of developing endometrial cancer. However, this risk is outweighed by the reduction in recurrence or in development of a contralateral breast cancer. Women on tamoxifen should be advised to report abnormal bleeding or vaginal discharge. Screening of asymptomatic women on tamoxifen therapy with ultrasound or endometrial biopsies is not currently recommended [18, 19]. This might become less of an issue over the next few years with a move to the use of aromatase inhibitors as a substitute for tamoxifen in the adjuvant treatment setting.

Offering screening for the group of patients with clear hereditary predisposition is equally challenging. Although most cases of endometrial carcinoma are thought to be sporadic, some cases clearly have a hereditary basis, the prototype being the Lynch syndrome (hereditary nonpolyposis colorectal cancer (HNPCC)). This is an autosomal-dominant cancer susceptibility syndrome associated with early-onset colon, rectal, ovary, small bowel, ureter/renal pelvis cancers, and endometrial cancer. The lifetime risk of endometrial cancer in Lynch syndrome women is 40% to 60%, a risk similar to that of developing colon cancer [20]. It is estimated that 2–5% of all endometrial carcinomas, and 10% of endometrial cancers in women younger than 50 years, are Lynch syndrome related [21].

Lynch syndrome results of a germ-cell line mutation in one of the DNA mismatch repair genes (*MSH2, MLH1, or MSH6) *[22]. Risk-reducing surgery (prophylactic hysterectomy and bilateral salpingo-oophorectomy) has been shown to be an effective strategy for preventing ovarian and endometrial and ovarian cancers in these women [23]. There is no uniform screening strategy for these women. Currently, the American Cancer Society recommends annual endometrial biopsies starting at age 35 for women known to have or be at risk for HNPCC and does not recommend ultrasound scans. However, the National Comprehensive Cancer Center (NCCC) guidelines state that annual endometrial biopsies may only be useful in select patients. The NCCC guidelines also state that transvaginal ultrasound for ovarian and endometrial cancer detection may be considered at the clinician's discretion.

The relationship between *BRCA1 and BRCA2 *genes mutations and the risk of endometrial cancer is controversial and less clear than the role of *BRCA1 and BRCA2 *in hereditary breast and ovarian/primary peritoneal cancers. Such an association has been suggested [24], and some studies have attempted to address this hypothesis but failed to demonstrate any increase in risk associated with *BRCA1* and *BRCA2* mutations compared to the general population [25, 26]. In one study of 199 Ashkenazi Jewish patients with endometrial cancer, the frequency of germ-cell line *BRCA1 and BRCA2 *mutations (3 per 199, 1.5%) in endometrial cancer patients was comparable to the baseline rate of 2% in the Ashkenazi population, suggesting no increased risk [25]. In another study, 857 known *BRCA1 and BRCA2* carriers aged 45 to 70 were followed over time for the development of endometrial cancer [26]. With an average length of followup of 3.3 years, six women developed endometrial cancer. Four of the six patients had used tamoxifen. Compared to the expected rate of endometrial cancer in a general population, *BRCA* carriers who did not receive tamoxifen did not have a significant increase in risk of developing endometrial cancer. Thus it seems that screening for endometrial cancer is not warranted in known *BRCA1* or *BRCA2* mutations carriers.

## 3. Determining the Surgical Procedure 

The standard treatment for endometrial carcinoma remains surgical and includes an initial exploration with collection of peritoneal fluid for cytologic evaluation (intraperitoneal cell washings), total extrafascial hysterectomy with bilateral salpingo-oophorectomy, and appropriate surgical staging in patients considered at risk for extrauterine disease.

## 4. The Role of Laparscopic Hysterectomy

Current evidence on the safety and efficacy of laparoscopic hysterectomy (including laparoscopic total hysterectomy and laparoscopically assisted vaginal hysterectomy) for endometrial cancer is adequate to recommend the use of this procedure. Patient selection for laparoscopic hysterectomy for endometrial cancer should be carried out by a multidisciplinary gynaecological oncology team, and advanced laparoscopic skills are required for this procedure.

In a meta-analysis of 3 randomised controlled trials (RCTs) including 359 patients with 38, 44, and 79 months of followup, respectively, there were no significant differences in survival between laparoscopic hysterectomy (LH) and abdominal hysterectomy (AH) [27, 28]. The overall survival rate was 92% (169/184) for patients in the LH group and 88% (154/175) for patients in the AH group (*P* = 0.976). The disease-free survival rate was 88% (161/184) for LH and 88% (154/175) for AH (*P* = 0.986). A nonrandomised comparative study of 309 patients reported 5-year overall survival rates of 98% for both LH and AH. The 5-year progression-free survival rate was 96% for patients after LH and 97% for patients after AH (*P* = 0.74) [29].

Similar rates of recurrence were noted for patients treated with LH or AH. Rates of recurrence after LH ranged between 9–20% compared to 12–18% after AH [30–32]. In one study, including 40 patients treated by LH, there was a single case of port-site recurrence (2.5%) after a median followup of 79 months [32]. The hospital stay after LH is statistically significantly shorter than after AH [30, 31], with the proportion of patients staying more than 2 days being significantly higher in the AH group [33]. Conversion from laparoscopy to laparotomy ranges from 0–26% with an average of 5–8% [30, 31, 33–36]. Similar rates of intraoperative complications have been reported for LH and AH, but significantly fewer postoperative complications for LH compared with AH [27, 28, 33].

The Laparoscopic Approach to Cancer of the Endometrium (LACE) trial is currently recruiting patients in Australia. The primary objective of this study is to assess disease-free survival at 4.5 years postoperatively for women with apparent stage I endometrial cancer, comparing patients who are randomised to receive total laparoscopic hysterectomy (TLH) and patients who are randomised to receive total abdominal hysterectomy (TAH). The estimated enrolment is 640 patients and the study is due to be completed in 2014. Several studies describe laparoscopic hysterectomy with robotic assistance.

## 5. Peritoneal Cytology Results

After omission from the 2009 FIGO staging for endometrial carcinoma, the need for and the significance of a positive peritoneal cytology result became controversial. However, positive washings are most common in patients with grade 3 histologic types, metastases to the adnexae, deep myometrial invasion, or positive pelvic or para-aortic nodes [37–43]. 

In a Gynaecologic Oncology Group (GOG) study of 697 patients with endometrial cancer, and with information on peritoneal cytologic results and adequate followup, disease recurred in 25 of 86 patients (29.1%) with positive washings, compared with 64 of 611 patients (10.5%) with negative washings [44]. In 17 of the 25 recurrences with positive washings, the recurrences were outside the peritoneal cavity. The relative risk of death for patients with positive cytologic washings was increased threefold [45].

Positive peritoneal cytology was reported in 8.3%, 12.1%, and 15.9% of stage I endometrial carcinoma patients with grades 1, 2, and 3 histologic types, respectively [46]. Superficial and deep myometrial invasion were associated with positive washings in 7.6% and 17.2% of the cases, respectively [46]. It might be concluded that the poor prognosis associated with malignant washings is a reflection of other adverse prognostic factors.

Positive peritoneal cytology may carry a prognostic significance only when the endometrial carcinoma has spread beyond the uterus [47–50]. In patients with clinical stage I and II endometrial carcinoma, positive peritoneal cytologic results did not influence survival when the disease was confined to the uterus [43]. However, when the disease had spread to the adnexa, lymph nodes, or peritoneum, then positive peritoneal cytologic findings decreased the survival rate from 73% to 13% at 5 years, but all recurrences were at distant sites [43]. Other studies have found that positive cytology was an independent poor prognostic factor for patients with stages I to IIIA disease [51, 52].

It could thus be concluded that the presence of positive peritoneal washings is not an independent negative prognostic indicator but potentiates other adverse prognostic indicators [42].

## 6. Surgical Staging and Lymphadenectomy

In addition, many women will require some type of adjuvant radiation therapy to help prevent vaginal vault recurrence and to sterilize disease in lymph nodes. With the increasing emphasis on surgico-pathologic staging, a more individualized approach to adjuvant radiation is possible. However, the need for pelvic and para-aortic lymphadenectomy remains a topic of heated debate.

There is a lack of consensus on the extent of surgical staging in endometrial carcinoma. The ability of surgical staging to accurately identify lymphatic spread and how this information affects prognosis and alters the use of adjuvant therapies are a source of controversy. When full surgical staging is performed, a bilateral pelvic and para-aortic lymphadenectomy is increasingly advocated on all endometrial carcinoma patients because positive lymph nodes (including isolated para-aortic lymph nodes) are common in all grades [53]. In the GOG 33 study, 22% of patients with clinical stage I and occult stage II endometrial carcinoma were found to have extrauterine spread at the time of surgery, with pelvic and/or para-aortic metastases in 11% of women [37]. Other studies have assessed various intraoperative parameters to identify patients having an extremely low probability of lymphatic spread in order to minimize under- and overtreatment [54, 55].

It is suggested that lymphadenectomy improves the carcinoma-related survival and the recurrence-free survival in high-risk endometrioid adenocarcinoma patients [56]. Conversely, lymphadenectomy does not appear to benefit patients with grade 1 and 2 endometrioid lesions with myometrial invasion <50% and primary tumor diameter <2 cm  [53, 57]. It thus seems that there is increasing evidence against the need to perform systematic lymphadenectomy in low-risk cases (grade 1 or 2 endometrioid tumors confined to the inner half of the myometrium) [53].

The debate exists first for the need for lymphadenectomy, its extent, and whether it incurs a therapeutic benefit.

### 6.1. No Lymphadenectomy

The majority of patients with endometrial carcinoma are at low risk for nodal disease at presentation, and treatment decisions can be based on final pathologic information. Thus if node dissection was to be performed, the majority of patients would be node negative and not gain any benefit [37, 54]. The Postoperative Radiation Therapy in Endometrial Cancer (PORTEC) trial evaluated patients with stage IC, grade 1; stage IB-C, grade 2; or stage IB, grade 3 (old FIGO staging system) who underwent hysterectomy *without* lymph node dissection and compared observation to postoperative pelvic radiation [58]. Favourable outcomes were observed both with and without radiation therapy (5-year survival rates of 85% observation, 81% with pelvic radiation) [58].

Analysis of women with stage I endometrial cancer in the US National Cancer Institute's Surveillance Epidemiology and End Results (SEER) database (1988 to 1993) showed that 5-year relative survival for patients without nodal dissection was 98% compared to 96% in those undergoing nodal dissection, thus suggesting no discernible benefit for nodal dissection [55]. It could be argued that the increased use of radiation in unstaged patients may produce similar outcomes to patients who are staged and who avoid radiation therapy. This was addressed by the randomized trial “A Study in the Treatment of Endometrial Cancer” (ASTEC) that randomized patients with endometrial cancer treated with hysterectomy to pelvic lymphadenectomy or not. Following surgery, patients with stage I-IIA disease were then randomized again to observation or pelvic radiation therapy if they had grade 3, serous, or clear cell histology; >50% myometrial invasion; or endocervical glandular invasion (stage IIA). The results suggested that there was no advantage for routine lymphadenectomy [57].

However, without the node dissection information, the surgeon must rely on uterine factors to estimate the probability for nodal disease and pelvic failure to determine the need for postoperative radiation, which could result in a substantial increase in the use of radiation and overtreatment of patients. Interestingly, omitting nodal dissection may also lead to poorer prognosis. For instance, a subset of 99 patients with stage IC, grade 3 endometrial cancer, who did not have lymph node dissection, were treated with pelvic radiation and followed prospectively within the PORTEC trial [59]. Five-year survival for this group of patients was 58%, and 12% had vaginal or pelvic failures despite whole pelvic radiation, which is poorer than what has been reported in patients with stage IIIC endometrial cancer managed by lymphadenectomy followed by radiation [60–62]. 

### 6.2. Selective Nodal Dissection

An alternative view is to reserve nodal dissection for patients with high risk of nodal disease. However, the risk of nodal disease that warrants the procedure is debated (3%, 5%, 10%, etc.). In endometrial cancer, major complication rates associated with nodal dissection are 2% to 6%, suggesting that this might be an appropriate level of risk to balance against the risk of nodal metastases. The GOG 33 study provides important data that can be used to decide whether to perform nodal assessments based on tumor grade and depth of invasion and frequency of nodal disease [37]. For example, the risk of pelvic nodal disease was 3% for all patients with grade 1 tumors, but was 11% with deeply invasive (outer one-third myometrial invasion) tumors. Patients with grade 3 tumors had a risk of pelvic nodal metastases of 18% and 34% with deep invasion. Patients with serous or clear cell histology also warrant nodal dissection as 30% to 50% will have nodal disease [63]. It seems that the depth of myometrial invasion is the most important factor that determines the likelihood of nodal involvement [37, 64, 65].

Selective lymph node dissection based on palpation is inaccurate as only 10% of patients with metastases to lymph nodes will have grossly enlarged nodes and frequently, even in these cases, direct palpation through the overlying peritoneum will fail to identify them [37].

Intraoperative assessment of the uterus has been used to guide the surgeon as to when to perform a nodal dissection, with gross inspection of the uterus immediately following its removal to estimate the degree of myometrial invasion. However, there is no typical gross appearance of an endometrial carcinoma. Frozen section assessment has been suggested as a tool to facilitate decisions on selective nodal dissections. Several studies have demonstrated inaccuracies with frozen sections in the interpretation of grade and depth of myometrial invasion compared to final pathology [66, 67]. In one prospective evaluation, frozen section determination of depth of invasion correlated with final pathology in 67% of cases but resulted in upstaging in 28% of cases [67].

### 6.3. Routine Lymphadenectomy

The strongest argument for routine staging is the avoidance of pelvic radiation therapy following thorough nodal assessment and confirmation of node-negative disease and low risk status. In the absence of nodal disease, recurrence risk is low and overall survival is high, with no radiation or with the substitution of vaginal vault brachytherapy. This has encouraged many gynaecologic oncologists to move towards performing routine surgical staging including pelvic and para-aortic lymphadenectomy for nearly all patients with endometrial cancer. 

The rationale for routine uniform staging is the inaccuracy of preoperative or intraoperative assessments predicting the risk for nodal disease, the potential for therapeutic benefit in node-positive and -negative patients, and the lack of *significant* morbidity associated with the procedure, with major complication rates of 2% to 6%. It could thus be argued that routine nodal dissection is the best method to determine which few patients will require adjuvant therapy. In addition, there is a significant risk of lymph node spread even for patients with seemingly low-risk disease. Data suggest that stage IA grade 1 disease has a 1% risk of para-aortic and a 2% risk of pelvic lymph node metastases, while stages IA grade 2 and IB grade 1 have a 2% risk of para-aortic and a 4% risk of pelvic lymph node metastases. Stage IB grade 2 disease has a 2% risk of para-aortic and a 6% risk of pelvic lymph node metastases [68].

However, a randomized controlled trial in which women with stage I endometrial cancer were assigned to have a standard hysterectomy and ovary removal with or without lymphadenectomy found that systematic use of pelvic lymphadenectomy does not improve disease-free or overall survival in women with early-stage endometrial cancer [69]. With a median followup of four years, there was no difference in patient outcomes between the two arms. Thirty-four (12.9%) of the 264 patients in the lymphadenectomy arm and 33 (13.2%) of the 250 patients in the control arm had disease recurrence. The median time to disease recurrence was 14 months in the lymphadenectomy arm and 13 months in the control arm. Overall five-year survival estimates were 86% for the lymphadenectomy arm and 90 percent for the nonlymphadenectomy. Surgical staging of the disease was improved with the systematic use of lymphadenectomy. A total of 13.3% of the women in the lymphadenectomy arm were found to have disease spread to pelvic lymph nodes, compared with 3.2% of the women in the control arm. The investigators found that although lymphadenectomy was not statistically significantly associated with improved survival, disease spread to the nodes was associated with poorer survival. Therefore, lymphadenectomy maintained its importance in determining a patient's prognosis and in tailoring adjuvant therapies [69].

## 7. Extent of Lymphadenectomy

The clinic-pathologic factor most strongly related to para-aortic nodal metastasis is pelvic lymph node metastasis [70]. Among patients who underwent systematic pelvic and para-aortic lymphadenectomy, 96.2% had negative para-aortic nodes when the pelvic nodes were negative. However, when the pelvic nodes were positive, 48% also had positive para-aortic nodes [70, 71]; hence, systemic para-aortic lymphadenectomy is advocated on all high-risk patients, or in patients with two or more positive pelvic lymph nodes [71, 72].

However, this is a major surgery to undertake in patients who are usually elderly and obese, with other comorbidities. An extensive para-aortic lymphadenectomy significantly increases operating time and blood loss and also increases postoperative morbidity, particularly lower limb lymphoedema (in about 20% of patients) [73]. Lymphoedema is often complicated by recurrent episodes of cellulitis. It could thus be argued that primary prevention of lymphoedema by selective use of pelvic lymphadenectomy and avoidance of systematic para-aortic lymphadenectomy is highly desirable.

## 8. Therapeutic Role of Lymphadenectomy

The therapeutic role of lymphadenectomy is less well understood, but its ability to modify adjuvant therapy is being increasingly accepted. The first suggestion of a therapeutic value for pelvic lymphadenectomy was by Kilgore et al. [74]. With a mean followup of 3 years, patients undergoing multiple-site pelvic node sampling had a significantly better overall survival as well as a better survival for both low-risk and high-risk groups. An explanation for the increased survival in low-risk groups may be the removal of unrecognized micrometastasis, which goes undetected by standard pathologic processing techniques. 

Conversely, the current available evidence from the ASTEC trial does not support the claim that lymphadenectomy in endometrial cancer is therapeutic [57]. Unfortunately, there are several reasons why the ASTEC trial could have failed to show improved overall survival with routine lymphadenectomy. The trial required only a pelvic lymphadenectomy and utilized a second randomization for pelvic radiation for disease characteristics, which, following a negative nodal dissection, is typically avoided. Likewise, vaginal vault radiation was permitted as per institutional practice irrespective of the assignment to pelvic radiation or not, making interpretations of any results likely difficult. The number of lymph nodes resected was insufficient in many patients. Equally, there was a high rate of inclusion of low-risk patients, and the low number of lymph nodes removed could be the reason for the low rate of involved lymph nodes seen in the lymphadenectomy group.

## 9. Predicting Nodal Disease

In an attempt to minimize the impact of lymphadenectomy and assist selective lymph node dissection, efforts have been paid towards predicting nodal disease using positron emission tomography (PET). It seems that 18F-FDG PET/CT is an accurate method for the presurgical evaluation of pelvic nodes metastases with high sensitivity, specificity, and positive predictive value (nearing 100%) [75, 76]. High negative predictive value (93.1–97.2%) may be useful in selecting patients who only may benefit from lymphadenectomy in order to minimize operative and surgical complications [77]. Conversely, other studies found that FDG-PET is only moderately sensitive in predicting lymph node metastasis preoperatively in patients with endometrial carcinoma with sensitivity and specificity of FDG-PET which were 60% and 98%, respectively [78, 79].

In an attempt to avoid complete lymphadenectomy, the concept of sentinel node identification has been investigated in endometrial carcinoma [80–87]. Data are scant, and studies are still addressing feasibility and standardization of technique. Many problems arise with the use of sentinel lymph node dissection. First, the lymphatic drainage of the uterus is considerably more complicated than that of the vulva and cervix. Second, there is no easily accessible or visible lesion in endometrial cancer as there is in vulvar or cervical cancers, making injection difficult. Third, the variation of reported locations of sentinel nodes ranges from the parametrium to the para-aortic region on either side of the body. These issues regarding the primary tumor and the patterns of lymphatic drainage make sentinel lymph node biopsy for endometrial carcinoma less practical.

## 10. Adjuvant Radiation Therapy

Radiation therapy has been crucial in the management of endometrial cancer, whether used as an adjuvant treatment after surgery or as definitive treatment for patients who are medically inoperable or with local recurrence.

The need for postoperative radiotherapy is usually determined by prognostic features obtained from the pathology review. Several studies suggested that survival rate increases if surgery is performed in conjunction with adjuvant pelvic radiotherapy, external beam radiotherapy (EBRT), or brachytherapy (BT) [88, 89]. Radiation therapy decreases the risk of pelvic recurrence [90]. Postoperative radiotherapy in women with stage II endometrial carcinoma patients led to an improved 5-year disease-free survival [91]. Similar results were observed in women with stage IIIC endometrial carcinoma receiving adjuvant EBRT and EBRT/BT. When direct extension of the primary tumor was present, the addition of BT to EBRT was even more beneficial [92]. Women with stage II endometrial carcinoma who did not receive radiation were 48% more likely to die from their tumours. The benefit of adjuvant radiation is most pronounced in women with high-risk pathologic features [93].

However, it seems that adjuvant pelvic radiotherapy leads to an improved local control but no overall survival advantage, particularly in low risk endometrial cancer patients [94]. Actually, in low-risk patients adjuvant EBRT is probably detrimental whilst for intermediate-risk patients although there may be a small benefit for some patients; this is offset by additional morbidity leading to an overall neutral effect [95–97]. Data from the ASTEC/EN.5 showed no evidence that overall survival with external beam radiotherapy was better than observation (hazard ratio 1.04; 95% CI 0.84–1.29). As such, adjuvant external beam radiotherapy cannot be recommended as part of routine treatment for women with intermediate-risk or high-risk early-stage endometrial carcinoma with the aim of improving survival [98]. In the PORTEC-1 trial, the 5-year risk of vaginal and pelvic recurrence for high/intermediate risk patients was 19% without further treatment, compared to 5% after EBRT.

Brachytherapy is perceived to be a more convenient mode of treatment compared to external beam radiotherapy and might be associated with less toxicity. PORTEC-2 compared the efficacy of vaginal BT and EBRT to determine which treatment provides optimal local control with best quality of life. The data suggested that vaginal brachytherapy is effective in preventing vaginal recurrence. Despite the slightly but significantly increased pelvic failure rate in the VBT arm, rates of distant metastases, OS, and RFS were similar [99]. Vaginal brachytherapy provided a better quality of life than external-beam radiotherapy for endometrial carcinoma and should be the preferred treatment from a quality of life perspective [100].

## 11. Adjuvant Chemotherapy

The value of adjuvant systemic chemotherapy in patients with high-risk early stage endometrial cancer is still controversial.

The GOG 34 trial, using single-agent doxorubicin, did not show any benefit women with clinical stage I or II (occult) disease who had one or more risk factors for recurrence after surgical staging [101]. Comparing 5 cycles of cisplatin, doxorubicin, and cyclophosphamide with external pelvic radiation, there was no difference between therapies in terms of progression-free or overall survival [102].

In patients with stages IC to IIIC endometrioid adenocarcinoma, there were no significant differences in disease-free or overall survival at a median followup of 5 years, between those treated with whole-pelvic irradiation or those who received three or more cycles of cyclophosphamide, doxorubicin, and cisplatin [103].

PORTEC-3 study is a phase III randomized trial comparing chemoradiation and adjuvant chemotherapy (4 cycles of carboplatin and paclitaxel) versus pelvic radiation alone in high risk and advanced stage disease. The study should determine whether radiotherapy or chemotherapy improves overall survival and failure-free survival, compare the rates of severe (grades 3 and 4) treatment-related toxicity, pelvic and distant recurrence, and evaluate quality of life of patients with high-risk and advanced stage endometrial carcinoma. 

Two additional GOG studies are currently open for recruitment and are examining the role of adjuvant chemotherapy in the treatment of endometrial carcinoma. The GOG 249 is a randomised phase III trial studying pelvic radiation therapy to see how well it works compared with vaginal implant radiation therapy, paclitaxel, and carboplatin in treating patients with high-risk stage I or stage II endometrial cancer. The GOG 258 is a randomised phase III trial comparing the recurrence-free survival of patients with stage I-IVA endometrial carcinoma treated with adjuvant chemoradiotherapy comprising cisplatin and tumor volume-directed radiotherapy followed by carboplatin and paclitaxel versus carboplatin and paclitaxel alone. The results of these trials are awaited by 2014.

## 12. Endometrial Carcinomas in Young Women

Less than 5% of endometrial cancers occur in women aged 40 years or younger, and the majority are well-differentiated tumours (90% of lesions) and are limited to the endometrium [104]. A more variable histologic pattern may occur in association with Lynch syndrome (HNPCC) [105]. Fertility preservation could be an issue in these patients and progestational therapy may be used. Endometrial carcinoma has been reported to progress in 80% of cases with various progestational agents (e.g., megestrol acetate 160–320 mg/day or medroxyprogesterone acetate 200–500 mg/day) [106–110]; however, successful pregnancies have been reported in only 40% of these women. A decision for uterine preserving treatment should not be taken lightly. It is crucial to exclude the presence of significant myometrial invasion by MRI and to ascertain a grade 1 histology and progesterone receptor positive status. Ovarian pathology has to be excluded in particular in association with Lynch syndrome [111] and to rule out synchronous primary or metastatic disease [112]. The endometrial response needs to be assessed after 3 months of progestational treatment. Unfortunately, about 40% of patients who initially respond will recur [106–110], hence the need for prolonged surveillance and the recommendation for hysterectomy once childbearing is completed.

## 13. Summary

Endometrial cancer is the most common gynecologic malignancy, and an understanding of presentation, surgical management, and treatment options is required for gynecologic oncologists. Surgical therapy is a mainstay of endometrial cancer treatment with lymphadenectomy and laparoscopy being increasingly integrated. A thorough knowledge of the relationships between uterine factors and extrauterine disease spread is essential. At present, surgical staging better defines extent of disease and largely defines risk of recurrence. Pelvic radiation is associated with better local control. In the published trials, there was no improvement in survival for selected patients with stage I-II endometrial cancer, as these trials did not include patients at high risk of recurrence and death. Chemotherapy is increasingly integrated into upfront management of advanced-stage endometrial cancer and may have a role in early-stage disease. Combination therapy with radiation and chemotherapy is under evaluation.
